# Characterization of driver mutations identifies gene signatures predictive of prognosis and treatment sensitivity in multiple myeloma

**DOI:** 10.1093/oncolo/oyae244

**Published:** 2024-09-09

**Authors:** Jian-Rong Li, Abinand Krishna Parthasarathy, Aravind Singaram Kannappan, Shahram Arsang-Jang, Jing Dong, Chao Cheng

**Affiliations:** Department of Medicine, Baylor College of Medicine, Houston, TX 77030, United States; Institute for Clinical and Translational Research, Baylor College of Medicine, Houston, TX 77030, United States; Department of Bioengineering, Rice University, Houston, TX 77005, United States; Department of Biology, Baylor University, Waco, TX 76706, United States; Division of Hematology and Oncology, Department of Medicine, Medical College of Wisconsin, Milwaukee, WI 53226, United States; Division of Hematology and Oncology, Department of Medicine, Medical College of Wisconsin, Milwaukee, WI 53226, United States; Medical College of Wisconsin Cancer Center, Milwaukee, WI 53226, United States; Linda T. and John A. Mellowes Center for Genomic Sciences and Precision Medicine, Medical College of Wisconsin, Milwaukee, WI 53226, United States; Department of Medicine, Baylor College of Medicine, Houston, TX 77030, United States; Institute for Clinical and Translational Research, Baylor College of Medicine, Houston, TX 77030, United States; The Dan L Duncan Comprehensive Cancer Center, Baylor College of Medicine, Houston, TX 77030, United States

**Keywords:** multiple myeloma, TP53, LRP1B, gene signatures, prognostic prediction

## Abstract

In multiple myeloma (MM), while frequent mutations in driver genes are crucial for disease progression, they traditionally offer limited insights into patient prognosis. This study aims to enhance prognostic understanding in MM by analyzing pathway dysregulations in key cancer driver genes, thereby identifying actionable gene signatures. We conducted a detailed quantification of mutations and pathway dysregulations in 10 frequently mutated cancer driver genes in MM to characterize their comprehensive mutational impacts on the whole transcriptome. This was followed by a systematic survival analysis to identify significant gene signatures with enhanced prognostic value. Our systematic analysis highlighted 2 significant signatures, TP53 and LRP1B, which notably outperformed mere mutation status in prognostic predictions. These gene signatures remained prognostically valuable even when accounting for clinical factors, including cytogenetic abnormalities, the International Staging System (ISS), and its revised version (R-ISS). The LRP1B signature effectively distinguished high-risk patients within low/intermediate-risk categories and correlated with significant changes in the tumor immune microenvironment. Additionally, the LRP1B signature showed a strong association with proteasome inhibitor pathways, notably predicting patient responses to bortezomib and the progression from monoclonal gammopathy of unknown significance to MM. Through a rigorous analysis, this study underscores the potential of specific gene signatures in revolutionizing the prognostic landscape of MM, providing novel clinical insights that could influence future translational oncology research.

## Teaser

This study aimed to enhance prognostic understanding of multiple myeloma by analyzing pathway dysregulations in key cancer driver genes to identify actionable gene signatures.

**Implications for practice**

Treatment strategies for multiple myeloma (MM) have not varied based on different clinical risk factors. Based on the insights derived from the TP53 and LRP1B gene signatures, our study underscores their utility in advancing MM management. Implementing these signatures as part of routine prognostic assessments could significantly enhance the precision of patient risk stratification. Additionally, by understanding individual responses to proteasome inhibitors, clinicians can tailor more effective, personalized treatment strategies, especially for high-risk patients. This approach also has potential implications for monitoring and intervening early in patients with monoclonal gammopathy of undetermined significance (MGUS), potentially preventing progression to MM.

## Introduction

Multiple myeloma (MM), a hematologic malignancy, arises from the aberrant proliferation of clonal plasma B cells within the bone marrow, leading to osteolytic lesions, anemia, and acute kidney injury.^1,2^ Despite advances in therapeutic strategies, MM remains an incurable disease,^3,4^ though it can be managed with existing treatments.^5,6^ Current treatment approaches for patients with MM generally follow a uniform protocol, irrespective of individual risk factors, or disease characteristics.^7-9^ Consequently, there is a growing need for improved prognostic risk stratification methods to tailor patient management more effectively, especially as the variety and complexity of available drug classes expand.^9^

Traditionally, prognostic risk in MM has been assessed using clinical factors, including serum molecule levels (eg, calcium, creatinine, c-reactive protein, hemoglobin, and albumin)^10,11^ and cytogenetic abnormalities (CAs).^9,12^ The International Staging System (ISS)^13^ and its Revised version (R-ISS)^14^ utilize these factors, such as serum β2-microglobulin, albumin, lactate dehydrogenase (LDH) levels, and specific CAs (eg, del(17p), t(4;14), t(14;16)), to more accurately estimate patient prognostic risk. However, there remains a need for further refinement in risk stratification,^15^ potentially incorporating gene-expression profile (GEP) based assessments^16-18^ or a Second Revision of the ISS (R2-ISS) that accounts for 1q gain and multiple CAs.^19^

Several driver genes, such as *KRAS*/*NRAS*, *TP53*, *BRAF*, and *CCND1*, frequently harbor mutations in MM.^20^ The mutation status of these genes is increasingly examined in clinical research for its potential in guiding personalized treatment.^20-22^ Nevertheless, the prognostic significance of these mutations is limited, as only a few are notably associated with prognosis. This limitation arises because prognosis is primarily influenced by oncogenic pathways related to these mutated genes, which can also be deregulated through other mechanisms like gene amplification/deletions, DNA methylations, and mutations in related or interacting pathways.^23-25^ Prior studies have suggested that gene signatures, encapsulating comprehensive regulatory information in the transcriptome of perturbed genes, serve as more effective prognostic markers than mutation status alone in various cancer types.^26,27^

In this study, we utilized mutation and expression data from the Multiple Myeloma Research Foundation (MMRF)-Clinical Outcomes in Multiple Myeloma to Personal Assessment of Genetic Profiles study (CoMMpass)^28^ to identify gene signatures reflective of the transcriptomic impacts of 10 frequently mutated cancer driver genes in MM. Our findings indicate that among these signatures, those of *TP53* and *LRP1B* are highly predictive in patients, regardless of mutation or cytogenetic abnormality status. Notably, the *LRP1B* signature offers insights into the immune microenvironment composition in MM, the response of patients to the proteasome inhibitor bortezomib, and the progression likelihood of Monoclonal Gammopathy of Undetermined Significance (MGUS) to MM. This study underscores the utility of gene signatures derived from driver somatic mutations in translational oncology research.

## Methods

### Datasets collection and process

Gene expression profiles, clinical information, and genomic variation data were sourced from several studies: CoMMpass,^28^ University of Arkansas for Medical Sciences (UAMS),^29^ MicroArray Quality Control II (MAQC),^30^ Assessment of Proteasome Inhibition for Extending Remissions phase 3 trial (APEX),^31^ Dutch-Belgian Cooperative Trial Group for Hemato-Oncology HOVON-65/GMMG-HD4 trail (HOVON65),^32^ Mayo Clinic Study (Mayo),^33^ Zhan Study (Zhan),^34^ and the Sun Study^35^ (Supplementary Table S1). For the CoMMpass study, expression profiling and SNV data were obtained from the Genomic Data Commons data portal (https://portal.gdc.cancer.gov/projects/MMRF-COMMPASS), and clinical data was downloaded from the dbGaP^36^ under the phs000748.v7.p4. Gene expression and clinical data for the other studies were obtained from the Gene Expression Omnibus (GEO) database^37^ under specific accession numbers: GSE136400, GSE24080, GSE9782, GSE19784, GSE6477, GSE5900, and GSE235356.

### Construction of gene-perturbed regulation signatures

To develop a signature for gene dysregulation based on transcriptome and pathway changes, we utilized SNV and expression data from the CoMMpass dataset. We selected 10 cancer census genes from the COSMIC database^38^: *KRAS*, *NRAS*, *LRP1B*, *MUC16*, *BRAF*, *FAT4*, *FAT3*, *CSMD3*, *FAT1*, and *TP53*, each mutated in at least 30 samples to ensure sufficient statistical power during the signature construction procedure (Supplementary Table S2). We assessed the impact of mutation status on gene expression using a linear regression model formulated as “gene expression ~ mutation status + age + gender” to calculate coefficients (𝛽-values) and *P*-values for each gene.

The resultant gene signature comprises 2 weight vectors: up-regulated weight (*w*^*up*^) and down-regulated weight (*w*^dn^). For genes where 𝛽 > 0, *w*^up^ is assigned as −log(*P*-value) and *w*^dn^ is set to 0. Conversely, for 𝛽 < 0, *w*^up^ is 0 and *w*^dn^ becomes −log(*P*-value). Weights exceeding 10 were capped at this value and subsequently normalized to range between 0 and 1, ensuring consistency before further analysis.

### Calculation of gene signature scores

To quantify gene dysregulation scores for all MM samples across various datasets, we employed a modified version of the gene set enrichment analysis method, Binding Associated with Sorted Expression (BASE),^39^ as described by previous studies.^26,27^ We first normalized gene expression profiles for each dataset by converting them into relative expression levels compared to the median expression across all samples for each gene. Each sample’s relative gene expression profile was then sorted from highest to lowest expression.

The BASE algorithm calculates gene signature scores using 2 cumulative distribution functions: a foreground function *f(i)* and a background function *b(i)* across the sorted expression profiles (*e*_1_, *e*_2_, *e*_3_, . . . , *e*_g_) of *n* genes. The calculations are as follows:


$$\begin{array}{l} f\left(i\right)=\;\;\Sigma {\;}_{j=1}^{i}\left|e_{j}w_{j}\right|/\;\Sigma {\;}_{j=1}^{n}\left|e_{j}w_{j}\right|,\;1\leq i\leq n \end{array}$$

$$\begin{array}{l} b\left(i\right)=\;\;\Sigma {\;}_{j=1}^{i}\left|e_{j}\left(1-w_{j}\right)\right|/\;\Sigma {\;}_{j=1}^{n}\left|e_{j}\left(1-w_{j}\right)\right|,\;1\leq i\leq n \end{array}$$

Here, *w* represents the weights from the gene dysregulation signatures matrix, with *w*^up^ for up-regulated and *w*^dn^ for down-regulated scores. The foreground function increases rapidly for genes with high relative expression levels and significant weight values, while the background function generally behaves oppositely. The preliminary score, representing the maximal deviation between these 2 functions, is normalized against the absolute value of the mean of a null distribution estimated from 2000 permutations. The final gene signature score for each patient is calculated by taking the difference between the normalized up-regulated and down-regulated scores.

### Survival analysis

Survival analysis was conducted using the R package “*survival*.”^40^ The univariable and multivariable Cox proportional hazard models were performed using the “coxph” function to estimate the effects of the TP53 or LRP1BSS on MM survival by calculating hazard ratios and 95% confidence intervals. According to the previous studies of GEP-based MM risk stratification methods,^16,18,41^ about 10%-30% of patients were identified as high-risk. Therefore, in this study, we assumed 20% of patients were high-risk in each dataset, and 0.8 quantile of scores was used as the cutoff to stratify the patients into “high” and “low” gene score groups. The overall survival (OS), event-free survival (EFS), and progression-free survival (PFS) for each group were analyzed using the Kaplan-Meier method and compared with the log-rank test via the “survfit” function. Kaplan-Meier survival plots were generated using the R package “*survminer*”^42^ to visually depict survival differences between groups.

### Cytogenetic abnormality selection for analysis

In the analysis of the CoMMpass dataset, patients with MM were evaluated for 13 types of cytogenetic abnormalities (CAs), determining presence (“Yes”), absence (“No”), or excluding from analysis if “Not Done” or missing. Certain CAs like *t*(8;14) and *t*(12;14) were excluded due to low sample sizes. Del(13) and del(17) were removed because they do not include specific chromosome arms, and del1q was excluded because it is not a relevant CA in MM. The study focused on comparing TP53SS or LRP1BSS scores in patients with or without CAs like *t*(4;14), *t*(6;14), *t*(11;14), *t*(14;16), *t*(14;20), del(13q), del(17p), and 1q gain using the Wilcoxon rank sum test to identify significant differences in gene scores.

### Inferring immune cell infiltration and expression profile in the tumor microenvironment

The UAMS dataset provided gene expression profiles of paired-matched tumor CD138 + cells (*E*^MM^) and whole bone marrow (WBM) (*E*^WBM^) samples from 401 patients. To assess the cellular composition of WBM samples, we applied an MM cells signature matrix, MGSM27,^43^ using the digital cell quantifier^44^ algorithm implemented by the R package “*ADAPTS*.”^43^ The proportion of the CD138+ cells (*P*^MM^) in each WBM sample was estimated as the sum of 4 cell catalogs: plasma cells, B cells memory, MM plasma cells, and PlasmaMemory.^29^ We adjusted the total immune cell proportion in the TIME for each patient to 100% after accounting for these CD138+ cell types.

To explore the relationship between TP53 or LRP1BSS and the TIME, we only focused on the immune cells with at least a 1% average proportion. Additionally, the expression profiles of the TIME of each patient were estimated by (*E*^WBM^*—E*^MM^* *× *P*^MM^)/(1—*P*^MM^). Spearmen correlation analysis was performed using the expression profiles of the TIME and the TP53 or LRP1BSS. The immune-related TIME genes whose expression correlated with gene scores were identified with ρ > 0.1 or <−0.1 and *P*-value < 0.01 and used for subsequent pathway enrichment analysis to explore the effect of gene scores on TIME.

### Treatment response prediction and analysis

The APEX dataset^31^ includes responses of 76 dexamethasone-treated and 186 bortezomib-treated patients with MM. Patients exhibiting any response (CR, PR, MR) were labeled as responders (1), and others as non-responders (0). We analyzed the relationship between gene scores and treatment response using multivariable logistic regression, adjusting for demographic and clinical factors. Density plots grouped patients by gene score levels to assess responder enrichment, tested using Fisher’s exact test. Spearman correlation and gene set enrichment analysis were performed to explore the biological implications of gene scores on treatment response.

### Gene ontology biological process and KEGG pathway enrichment analysis

The R package “*clusterProfiler*”^45^ was utilized to perform enrichment analyses, using “enrichGO” for gene ontology (GO) biological process enrichment and “gseKEGG” to identify significant KEGG pathways correlated with gene scores. Enrichment was considered significant with an FDR < 0.05. Biological processes in TIME genes correlating with TP53 or LRP1BSS in CD138+ cells were investigated, and GSEA plots for KEGG results were generated using “gseaplot2.”

## Statistical analysis

The built-in R package “*stats*” was used for most statistical analyses, including demographic and score comparisons between groups using the “wilcox.test” for the Wilcoxon rank-sum test. The “p.adjust” function applied the Benjamini–Hochberg procedure for multiple testing adjustments. Statistical significance was set at *P*-values less than .05. All analyses were performed in R version 4.0.2.

## Results

### TP53 and LRP1B gene signatures predict prognosis in MM

**Figure 1.**
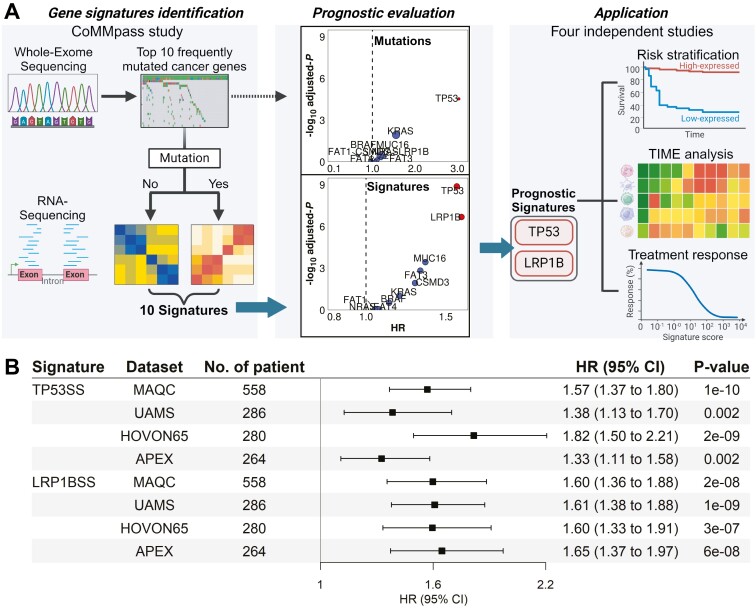
The overview of the study. (A) The expression profile and mutation profile of the CoMMpass data were used to generate the gene expression signatures of the top 10 most mutated driver genes. The expression profiles of patients with MM in each dataset were applied to the gene expression signature matrix to calculate the gene signature score of every patient. The Cox model results of the mutation status of the 10 most frequently mutated cancer driver genes in the CoMMpass dataset show that the mutation status of the *TP53* gene is the only gene with an MM prognosis prediction effect. In comparison, the TP53 signature was the most prognosis predictive, followed by the LRP1B signature. The TP53 and LRP1B signatures were used to evaluate the prognostic risks, investigate the interaction with the tumor immune microenvironment, and predict the treatment response of patients with MM. This figure was created with BioRender.com. (B) The prognosis evaluation for overall survival (OS) with the TP53 signature score (TP53SS) and LRP1B signature score (LRP1BSS) using 4 independent datasets, the TP53SS and LRP1BSS were predictive in all datasets.

Figure 1A shows an overview of this study, which used the CoMMpass dataset containing matched somatic mutation and gene expression profiles from CD138+ cells of 762 patients with MM. We developed gene signatures for the 10 most frequently mutated cancer genes^38^ (with non-synonymous mutations in ≥30 samples). These signatures calculated patient-specific scores that reflect pathway activities altered by mutations (Figure 1A).

Previous studies^26,27,46^ indicate that these signature scores provide deeper insights than mutation statuses alone, capturing pathway inactivation and over-activation due to alternative mechanisms. Survival analysis revealed that the TP53 and LRP1B signatures are strongly prognostically significant. Notably, mutation statuses of these genes typically showed weak associations with patient OS, with the exception of TP53 (Figure 1A).

These signatures were validated in 4 independent MM gene expression datasets: MAQC,^30^ UAMS,^29^ HOVON65,^32^ and APEX^31^ (Supplementary Table S1), comprising 1388 patients, showing significant correlations of TP53 signature score (TP53SS) and LRP1B signature score (LRP1BSS) with OS (Figure 1B) and PFS/EFS (Supplementary Figure S1A). Subsequent analyses investigated their prognostic value, interaction with the tumor immune microenvironment, and impact on patient responses to specific therapies (Figure 1A).

### Enhanced prognostic value of TP53 signature over mutation status and del(17p) in MM

**Figure 2.**
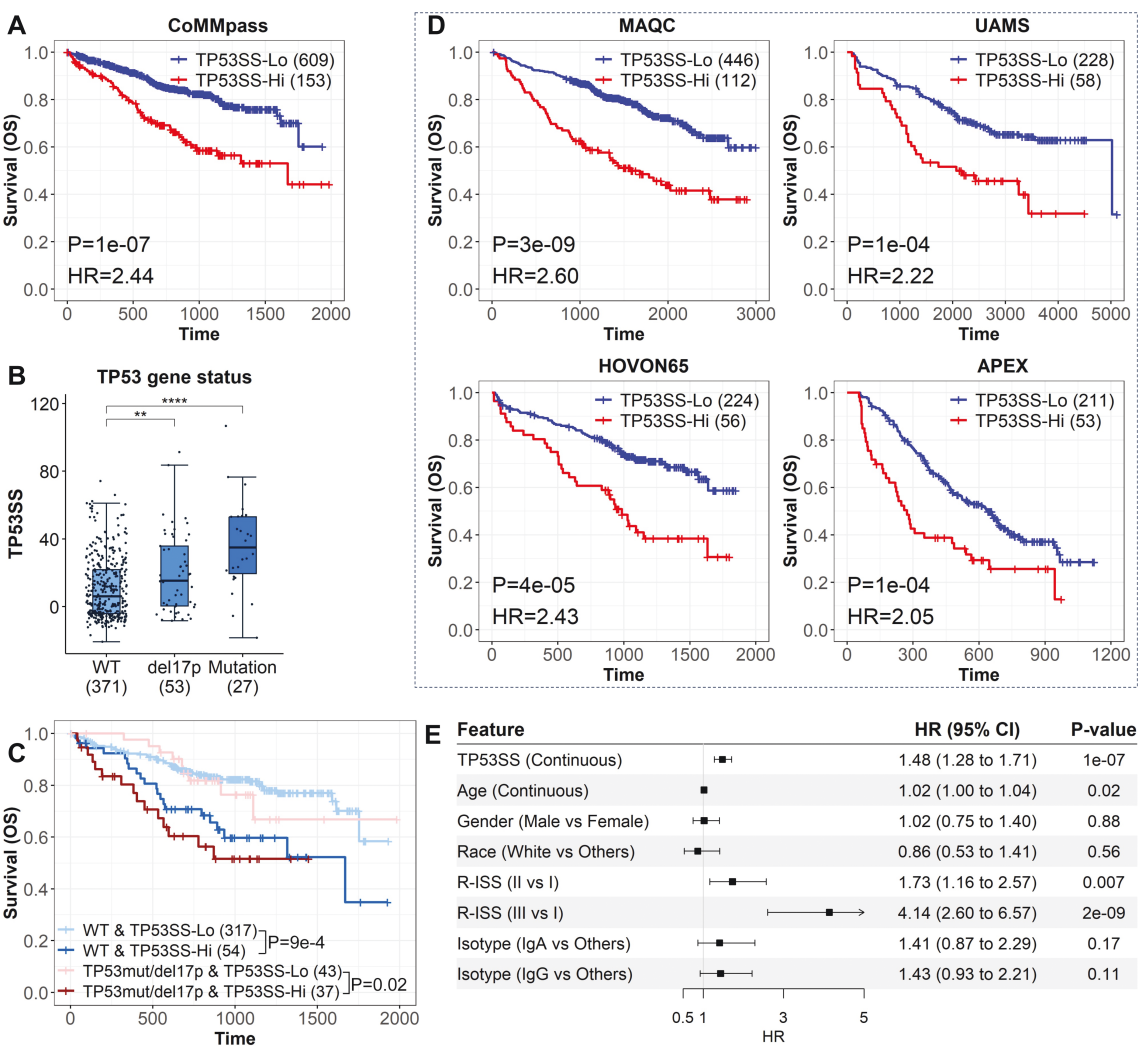
The TP53SS is prognosis predictive for patients with MM. (A) Overall survival curves of patients with MM with higher TP53SS (greater or equal to the 0.8 quantiles of the TP53SS) vs those with lower TP53SS in the CoMMpass dataset. Patients with MM with higher TP53SS have a significantly poorer prognosis. (B) TP53SS of patients with wild-type (WT), *TP53* mutations, and del(17)p, respectively. Patients with *TP53* mutation or del(17)p have significantly higher TP53SS than WT patients. **: *P* < .01, ****: *P* < .0001. (C) Overall survival curves for patients with higher TP53SS vs those with lower TP53SS in the groups that have been stratified by *TP53* mutation and del(17p) status in the CoMMpass dataset. (D) Overall survival curves of patients with MM with higher TP53SS vs those with lower TP53SS in the MAQC, UAMS, HOVON65, and APEX datasets. patients with MM with higher TP53SS have a significantly poorer prognosis. (E) The result of multivariable-adjusted Cox proportional hazards (PH) models of overall survival with age, gender, race, isotype, R-ISS stage system, and the TP53SS.

Among the 10 genes most frequently mutated in MM, only *TP53* mutations significantly impacted prognosis, with these mutations linked to poorer survival outcomes (*P* = 1e−4, HR = 2.89, Supplementary Figure S2A). Conversely, del(17p)—typically linked to loss of *TP53* function^47^—did not show significant prognostic influence in the CoMMpass dataset (*P* > 0.1, Supplementary Figure S2B). The TP53 signature, indicative of pathway disruptions, was more prognostically significant than mutation status alone, with higher TP53SS associated with worse OS (*P* = 1e−7, HR = 2.44, Figure 2A). Additionally, in the multivariable Cox regression results, TP53SS was a more significant predictor of prognosis than *TP53* mutation (Supplementary Figure S2C and D). Patients with del(17p) or *TP53* mutations also exhibited higher TP53SS compared to those with wild-type chr17p and *TP53* (Figure 2B), suggesting that TP53SS captures effects beyond TP53 mutations, including del(17p).

Stratification of patients into wild-type and mutant/deleted groups based on *TP53* mutation and chr17p status allowed TP53SS to further classify patients into subgroups with differing survival outcomes in both categories (Figure 2C), highlighting its superiority as a prognostic biomarker over *TP53* mutation and chr17p status.

Validation in 4 additional datasets confirmed that higher TP53SS consistently predicted worse OS (*P* < 0.05, HR > 1, Figure 2D) and PFS/EFS (*P* < 0.05, HR > 1, Supplementary Figure S2E). Furthermore, even after adjusting for clinical features such as Age, Gender, Race, Isotype, and R-ISS in the MAQC dataset, TP53SS remained highly predictive (*P* = 1e−7, Figure 2E), demonstrating its utility in providing additional prognostic information alongside clinical factors.

### The LRP1B signature provides additional prognostic values and links to pre-MM development

**Figure 3.**
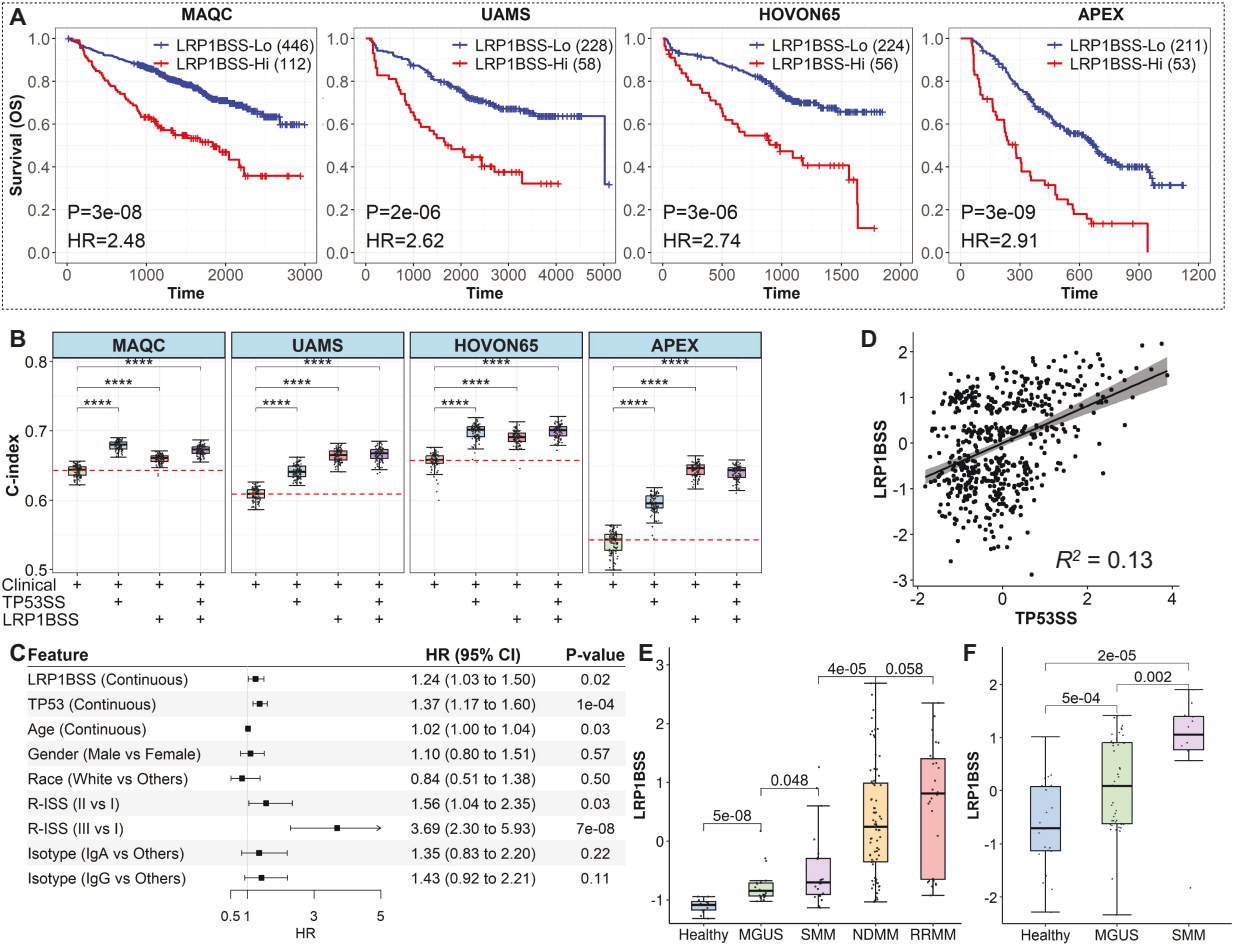
The LRP1BSS predicts the prognosis of patients with MM in addition to the TP53SS. (A) Overall survival curves of patients with MM with higher LRP1BSS vs those with lower LRP1BSS in the MAQC, UAMS, HOVON65, and APEX datasets. Patients with MM with higher LRP1BSS have a significantly poorer prognosis. (B) The C-index of the Cox PH prognosis model of OS using the available clinical factors, clinical factors with TP53SS, clinical factors with LRP1BSS, and clinical factors with TP53SS and LRP1BSS in 4 independent datasets. ****P < .0001. (C) The result of multivariable-adjusted Cox proportional hazards (PH) models of overall survival with age, gender, race, isotype, R-ISS stage system, and both TP53SS and LRP1BSS in the MAQC dataset. (D) The Spearman correlation between the TP53SS and LRP1BSS in the MAQC dataset. (E) The LRP1BSS between healthy controls, patients with monoclonal gammopathy of unknown significance (MGUS), smoldering multiple myeloma (SMM), newly diagnosed multiple myeloma (NDMM), and relapsed or refractory multiple myeloma (RRMM) in the Mayo dataset. (F) The LRP1BSS between healthy controls, patients with MGUS, and patients with SMM in the Zhan dataset.

In addition to the TP53 signature, preliminary analysis identified an LRP1B signature (Figure 1A and B). Across 4 validation datasets, higher LRP1BSS correlated with significantly shorter OS (*P* < 0.05, HR > 1, Figure 3A) and EFS/PFS (*P* < 0.05, HR > 1, Supplementary Figure S3A). Adjustments for clinical covariates using MAQC data showed LRP1BSS strongly correlated with prognosis (*P* = 6e−5, Supplementary Figure S3B). Adding TP53SS or LRP1BSS to clinical prediction models through 100 iterations of 5-fold cross-validation notably improved OS, PFS, and EFS prediction accuracy (Figure 3B and Supplementary S3C).

Both LRP1BSS and TP53SS remained significant after adjusting for each other and clinical variables (*P* = 0.02 and 1e−4 respectively, Figure 3C), indicating orthogonal prognostic capabilities in MM. Partial correlation between TP53SS and LRP1BSS (*R*^*2*^ = 0.13, Figure 3D) suggests independent variability. Analysis of TP53SS and LRP1BSS with GSVA scores^48^ of canonical pathways^49^ showed high correlation of TP53SS with cell cycle and DNA stability pathways, including Aurora B, PLK1, ATR, Fanconi, and E2F (*ρ* > 0.5, Supplementary Figure S3D), whereas LRP1BSS was linked to metabolic pathways of pyrimidine, purine, non-coding RNA, and mRNA (*ρ* > 0.5, Supplementary Figure S3E). This result implies that TP53SS captures the prognostic information most relevant for cancer progression, while LRP1BSS characterizes several regulatory abnormalities other than cancer progression.

LRP1BSS showed significant increases across most MM development stages, notably between monoclonal gammopathy of unknown significance (MGUS), smoldering multiple myeloma (SMM), and newly diagnosed multiple myeloma (NDMM), and nearly significantly from NDMM to relapsed or refractory multiple myeloma (RRMM) in the Mayo data^33^ (Figure 3E) and the Zhan dataset^34^ (Figure 3F). TP53SS increased significantly in NDMM and RRMM but was not associated with the pre-MM developmental stage (Supplementary Figure S4A and B). Analysis of commercial gene expression signatures, GEP70^18^ and SKY92,^17^ revealed no significant differences between MGUS and SMM, nor between NDMM and RRMM in the Mayo dataset (Supplementary Figure S4C), but a gradient increase in the Zhan dataset (Supplementary Figure S4D). In contrast, SKY92 showed a significant rise in RRMM compared to NDMM, with no clear trends during earlier MM stages in both datasets (Supplementary Figure S4E and F). These findings suggest that the LRP1B signature could predictively mark the early stages of MM development.

### LRP1B and TP53 signatures are prognostic in MM subgroups identified by clinical factors

**Figure 4.**
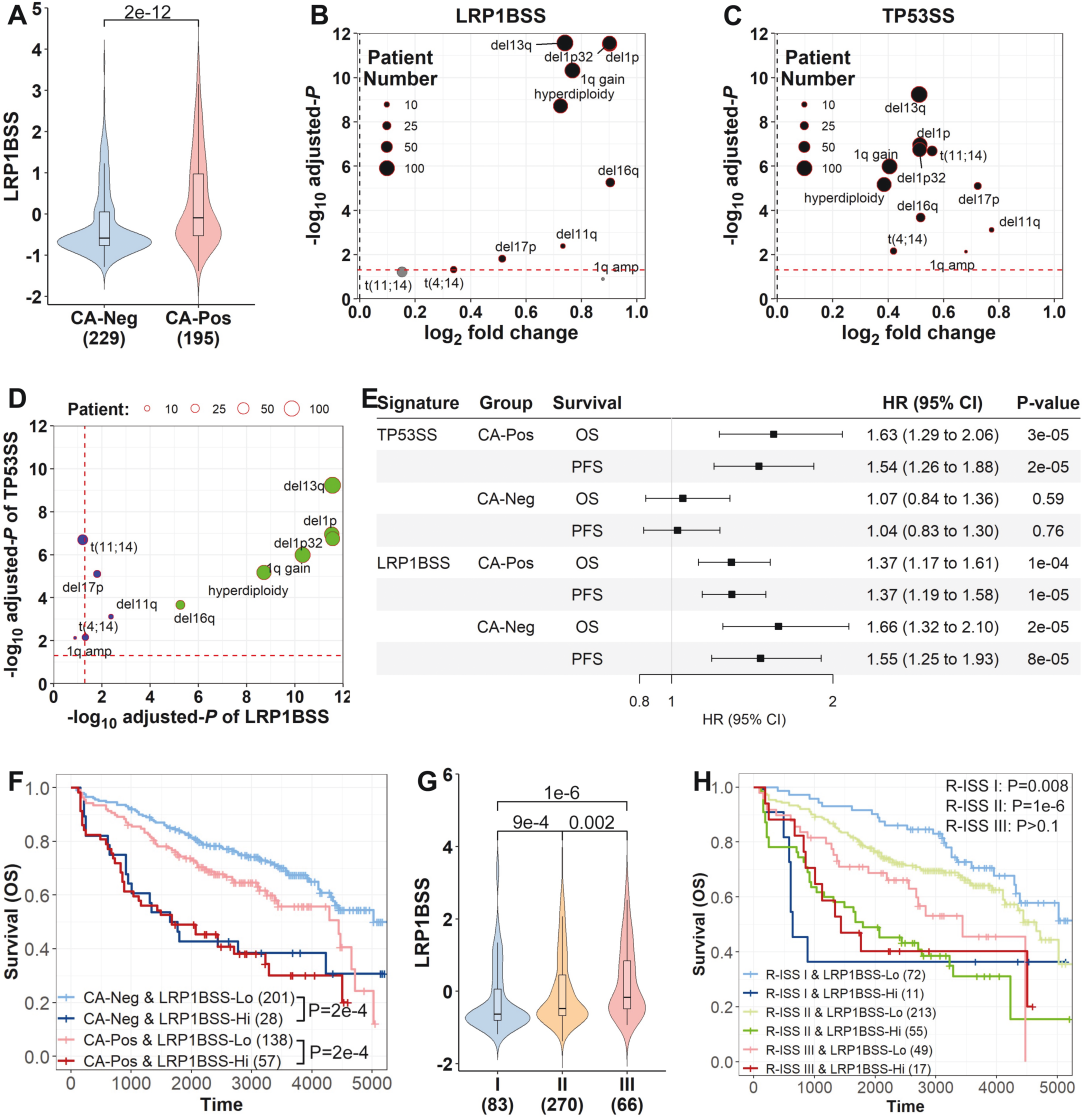
The TP53 and LRP1BSS provide additional prognosis information beyond the cytogenetic abnormalities. (A) LRP1BSS between patients with MM with and without cytogenetic abnormalities (CAs). Patients with CAs have higher LRP1BSS. (B) LRP1BSS between patients with MM with and without 12 types of tested cytogenetic alterations. Patients with CAs other than *t*(11;14) and 1q amplification had higher LRP1BSS. (C) TP53SS between patients with MM with and without 12 types of tested cytogenetic alterations. Patients with each type of CAs had higher TP53SS. (D) Comparison of the significance levels of 12 types of CAs between LRP1BSS and TP53SS. The CAs t(11;14), del17p, del11q, t(4;14), and 1q amplification are more significant associated with TP53SS, whereas the others are more significantly related to LRP1BSS. (E) Continuous values of TP53SS and LRP1BSS predict the prognosis of OS or PFS in patients with any CA (CA-Pos) or without any CA (CA-Neg). (F) Overall survival curves of patients with MM with high or low LRP1BSS in the groups of patients with any CA (CA-Pos) or without any CA (CA-Neg). Patients with higher LRP1BSS have poorer prognoses in both groups. (G) LRP1BSS between patients with MM with the R-ISS stages I, II, and III. Patients with higher R-ISS have higher LRP1BSS. (H) Overall survival curves of patients with MM with higher and lower LRP1BSS that belong to R-ISS I, II, and III. Patients with higher LRP1BSS had poor outcomes in R-ISS I and II but not in III. All results are based on the UAMS dataset. Significant level was calculated using the Wilcoxon rank sum test.

Patients with MM with CAs typically have a poorer prognosis than those without. Using the UAMS dataset, we found both LRP1BSS and TP53SS were significantly higher in patients with CAs (*P* = 2e−12 and 3e−11, respectively; Figure 4A and Supplementary Figure S5A), indicating these signatures capture CA-related prognostic information. LRP1BSS varied significantly across different CA categories, with pronounced differences in high-risk CA like Del(1p) compared to lower-risk CAs, while TP53SS was notably higher in all CA categories (Figure 4B-D). Notably, both LRP1BSS and TP53SS provide significant prognostic information after adjusting for their significantly related CAs (Supplementary Figure S5B-D).

We grouped patients into CA-positive (CA-Pos) and CA-negative (CA-Neg) categories, finding that both LRP1B and TP53 signatures significantly predicted prognosis in CA-Pos groups (Figure 4E). Notably, the LRP1B signature also significantly predicted prognosis in CA-Neg patients. Higher LRP1BSS was associated with poorer OS across both groups (Figure 4F), whereas TP53SS correlated with worse OS primarily in CA-Pos patients (Supplementary Figure S6A). Both signatures showed significant correlations with PFS as well (Supplementary Figure S6B and C).

Moreover, both LRP1BSS and TP53SS significantly increased with R-ISS stage, reflecting their capacity to capture R-ISS-related prognostic information (Figure 4G and Supplementary S6D). Within R-ISS stage groups, both signatures were significantly or near-significantly associated with OS and PFS outcomes (Supplementary Figure S6E). Specifically, high LRP1BSS in R-ISS I and II patients predicted significantly poorer OS, notably in R-ISS II, but not in R-ISS III (Figure 4H). In contrast, high TP53SS predicted significantly worse OS across all R-ISS groups, with lower significance in R-ISS II (Supplementary Figure S6F), and a similar pattern was observed for PFS (Supplementary S6G and -H).

### LRP1B signature is associated with differential tumor immune microenvironment

**Figure 5.**
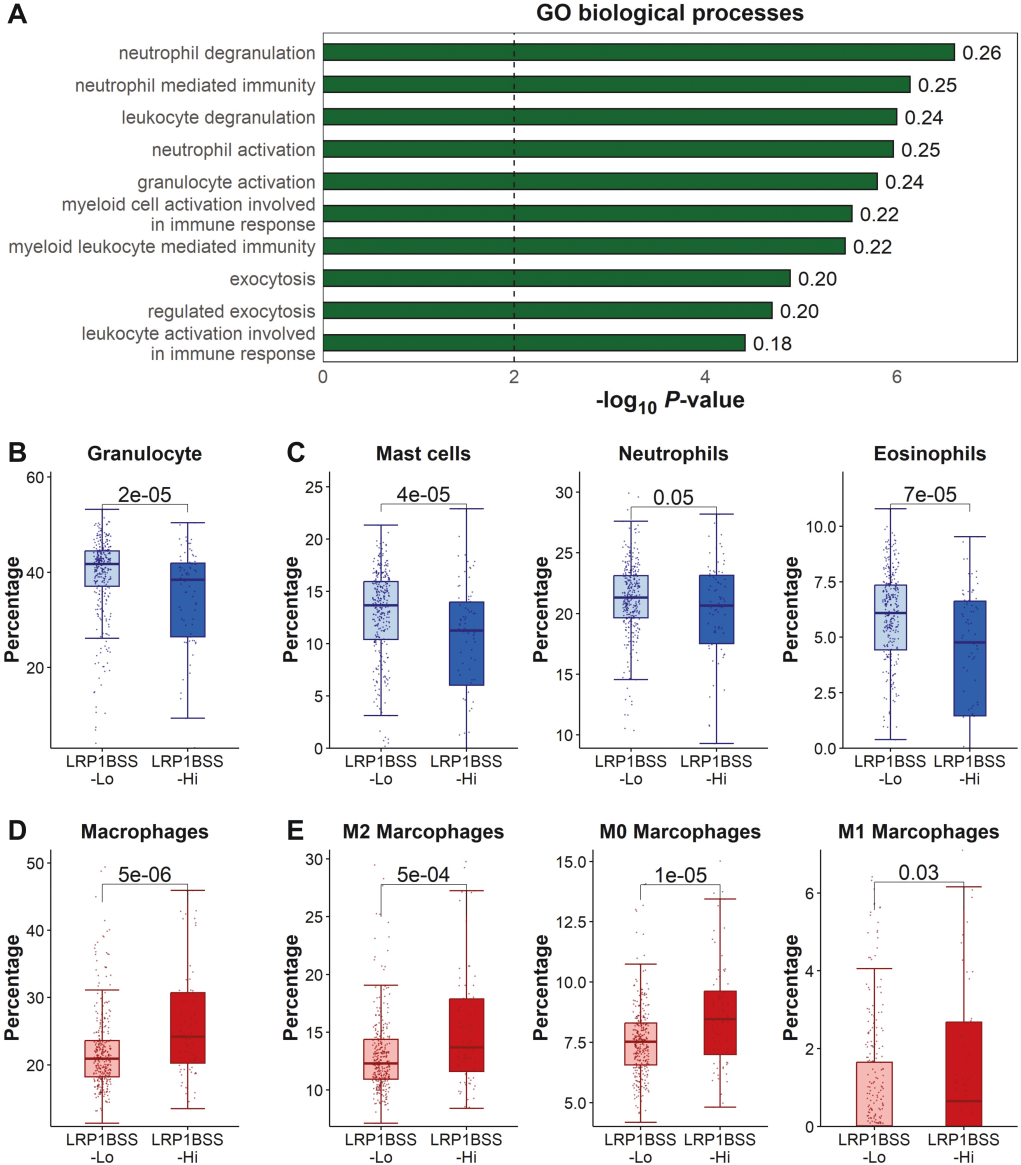
The LRP1BSS of CD138+ cells are related to the immune cell infiltration in the tumor immune microenvironment. (A) The biological processes of GO term that were enriched by the immune-related TIME genes negatively correlated with the LRP1BSS of the CD138 + cells. The cutoff is FDR < 0.05. The number after each bar is the gene ratio. (B) The percentage of the infiltrated granulocytes between patients with higher or lower LRP1BSS. Patients with higher LRP1BSS have significantly higher infiltrated granulocytes in the TIME. (C) The proportion of the infiltrated neutrophils, eosinophils, and mast cells between patients with higher or lower LRP1BSS. (D) The percentage of the infiltrated macrophages between patients with higher or lower LRP1BSS. Patients with higher LRP1BSS have significantly higher infiltrated macrophages in the TIME. (E) The percentage of the infiltrated M2, M0, and M1 macrophages between patients with higher or lower LRP1BSS. The significant level was calculated using the Wilcoxon rank sum test.

The UAMS dataset provides matched CD138+ cells and whole bone marrow (WBM) expression profiles for 401 MM samples, enabling us to investigate the interaction between malignant plasma B cells and the TIME (tumor immune microenvironment). According to the study by Danziger et al,^29^ we performed a deconvolution analysis to calculate the relative abundance of 24 different immune cell types using the whole bone-marrow expression data. Based on the expression profiles and the estimated proportion of CD138+ cells, we calculated the expression of 1397 immune-related genes in TIME as described in Methods. We identified 456 and 307 TIME immune-related genes with a significant correlation (*P *< .01) with the TP53SS and LRP1BSS in CD138+ cells, respectively. We found that the TIME genes that were negatively correlated to LRP1BSS were enriched in the GO biological processes of the immune response, activation, exocytosis, and degranulation of granulocytes (Figure 5A), suggesting the higher LRP1BSS in CD138+ cells are associated with the potential loss of granulocytes and thus immune function in TIME.

Indeed, the comparative analysis indicated that patients with higher LRP1BSS in CD138+ cells tend to have a significantly lower infiltrated level of granulocytes (Figure 5B), such as mast cells, neutrophils, and eosinophils (Figure 5C), in their TIME. Furthermore, patients with higher LRP1BSS have an elevated level of infiltrated macrophages (Figure 5D), including a significant increase in M0 macrophages, which represents undifferentiated and resting macrophages, as well as M2 macrophages known for their diverse tumor-promoting functions,^50^ and a slight increase in M1 macrophages (Figure 5E). These results suggest that the prognostic information provided by the LRP1B signature for patients with MM may partly capture the composition information of the TIME.

### The LRP1B signature predicts response to bortezomib

**Figure 6.**
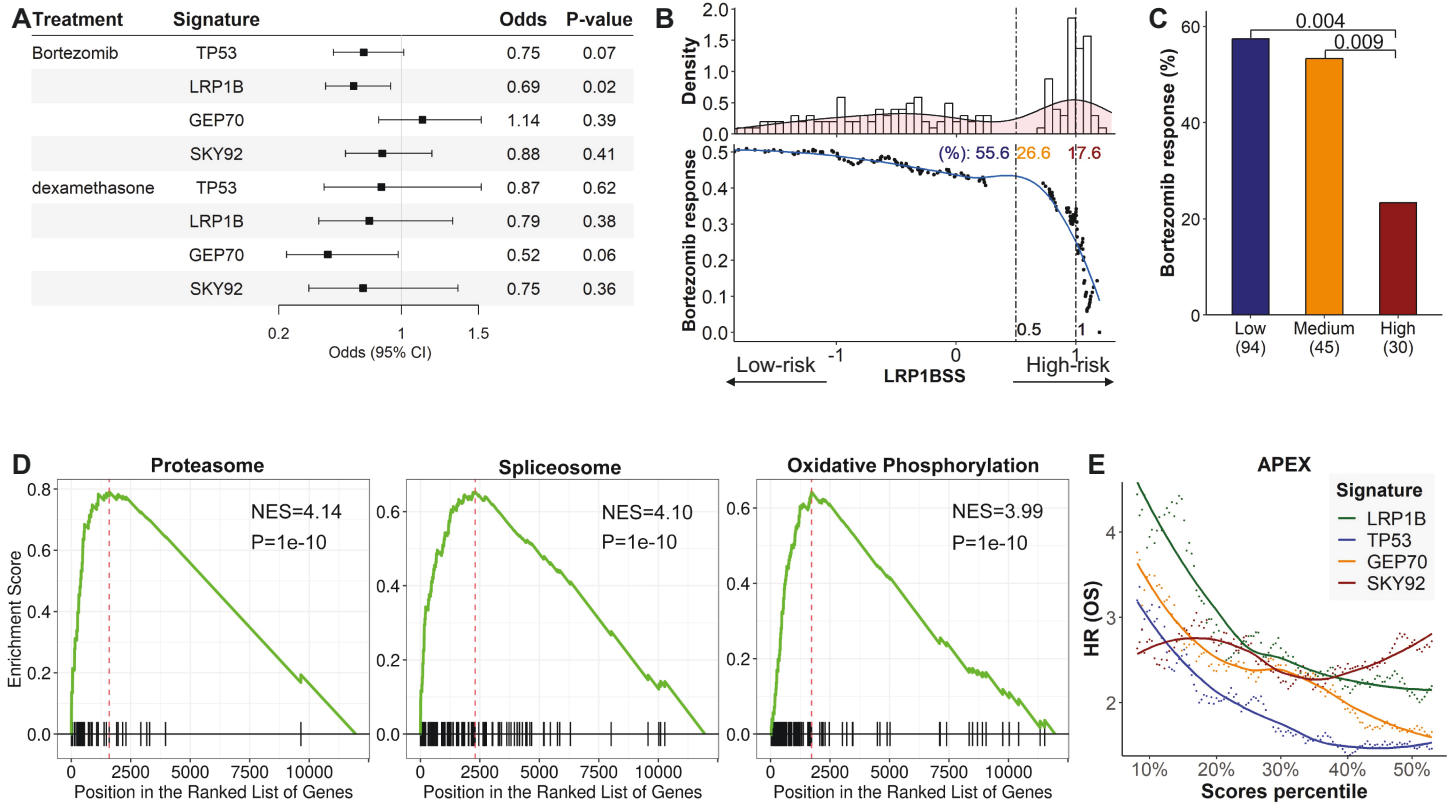
The LRP1BSS are related to the response of bortezomib, a proteasome inhibitor. (A) The results of using multivariable logistic regression to predict the drug response to bortezomib and dexamethasone using TP53SS, LRP1BSS, GEP70 score, and SKY92 score indicated a significant association between higher LRP1BSS and poorer bortezomib response in patients with MM. (B) The distribution of LRP1BSS and the response rates to bortezomib in patients with MM with LRP1BSS scores exceeding the specific cutoff belong to each patient. The 2 dotted lines indicate the numerical cutoffs of 0.5 and 1, respectively. The percentage is the proportion of patients in the group. (C) The percentage of bortezomib treatment responders in patients with MM with low, medium, and high LRP1BSS. Patients with high LRP1BSS have significantly lower bortezomib responder fraction. Fisher’s exact test. (D) The first 3 most enriched KEGG pathways, proteasome, spliceosome, and oxidative phosphorylation using the GSEA analysis. (E) RMM patients selected by LRP1BSS (top f% with highest scores) showed a higher hazard ratio (HR) relative to the rest patients than those selected by the other 3 signatures.

In our study using the APEX dataset, we evaluated the predictive power of LRP1BSS, TP53SS, GEP70, and SKY92 for patient responses to bortezomib and dexamethasone. Multivariate logistic regression incorporating age, gender, ethnicity, and ISS stage showed no significant associations with dexamethasone response for any signatures, but higher LRP1BSS scores were significantly linked to poorer bortezomib responses (Figure 6A). Response rates to bortezomib decreased as LRP1BSS scores exceeded 0.5, with a marked decline beyond a score of 1 (Figure 6B). Patients were grouped by LRP1BSS scores into high, medium, and low categories (cutoffs at 0.5 and 1), with the low and medium groups showing 2.5 and 2.3 times higher response rates than the high group (*P* = .004 and .009, respectively, Fisher’s exact test, Figure 6C).

Additionally, Spearman correlation was performed to analyze between LRP1BSS and gene expressions, accompanied by gene set enrichment analysis (GSEA)^49^ using KEGG pathways.^51^ The top 3 pathways significantly enriched with LRP1BSS were the proteasome, spliceosome, and oxidative phosphorylation pathways (Figure 6D), with citrate cycle/TCA cycle and protein export also showing substantial enrichment, ranking 14th and 28th, respectively (Supplementary Figure S7A and B). These pathways are known to be implicated in the action mechanism of the proteasome inhibitor bortezomib.^52-58^ This supports LRP1B’s role in predicting bortezomib resistance.

Given the predictive validity of LRP1BSS for drug response, we hypothesized its potential in predicting the overall survival in patients with RMM. By stratifying patients into 2 cohorts based on each signature, encompassing the highest and lowest f% (with *f* being a variable factor), their OS was compared to derive the HR between 2 groups. LRP1BSS demonstrated better efficacy compared to other signatures; at equivalent *f* values, the high-risk group delineated by LRP1BSS exhibited elevated HR values relative to those determined by other signatures, up to an *f* value of 50% (Figure 6E). Notably, LRP1BSS proved adept not only in identifying patients with an elevated risk of overall survival under bortezomib treatment (Supplementary Figure S7C) but also showed commendable performance in patients treated with dexamethasone (Supplementary Figure S7D).

### The LRP1B signature predicts the outcome of the MGUS progression

**Figure 7.**
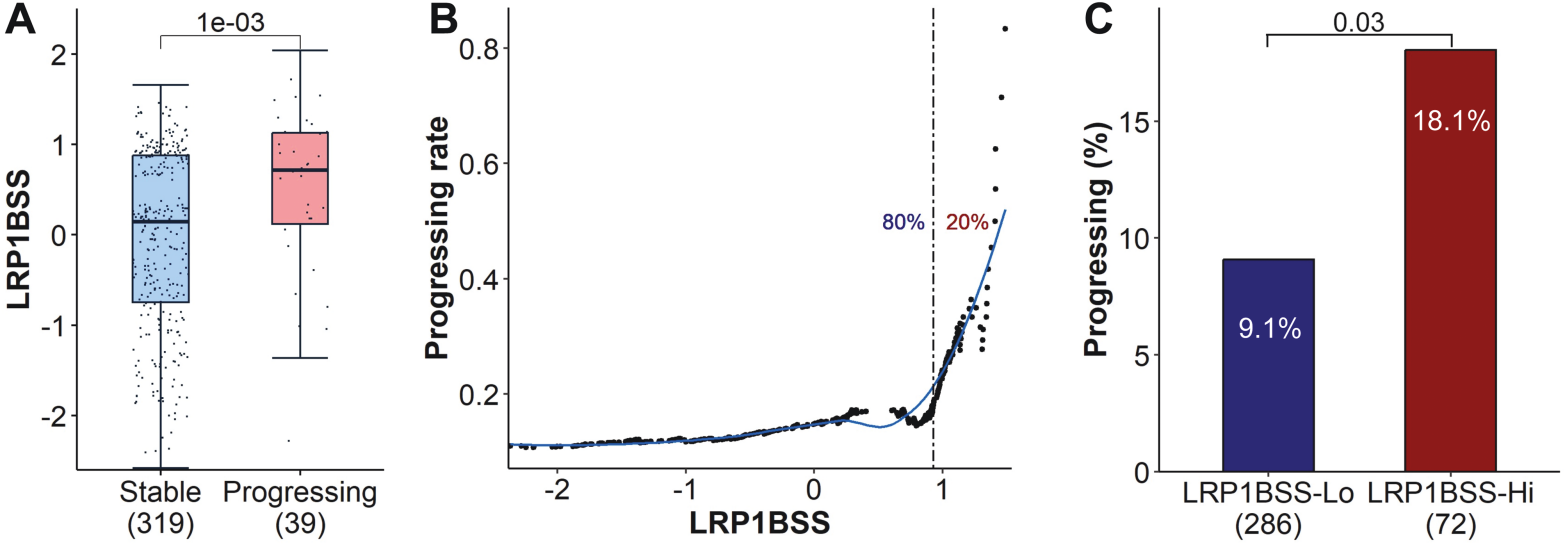
The LRP1BSS can predict the progression outcome of patients with MGUS. (A) LRP1BSS in MGUS patients who progressed to MM (Progressing) and those who remained stable (Stable). Patients in the progressing group exhibited significantly higher LRP1BSS. *P*-value calculated using the Wilcoxon Rank-Sum test. (B) Proportion of progressing MGUS patients with LRP1BSS exceeding the cutoff, determined individually for each patient. The dashed line represents the cutoff for the top 20% of patients with high LRP1BSS. (C) Pssroportion of progressing patients among groups with high and low LRP1BSS. *P*-value calculated using Fisher’s exact test.

In our previous findings, we showed a notable increasing of LRP1BSS across successive stages of pre-MM development (Figure 3E and F). This observation led us to hypothesize that LRP1BSS might possess prognostic utility in predicting the progression outcome of pre-MM stage evolution. To substantiate this hypothesis, we leveraged the dataset recently published by Sun et al^35^ This dataset encompasses gene expression profiles of 358 individuals diagnosed with MGUS, coupled with longitudinal data delineating their progression to MM, including 319 individuals exhibiting disease stability and 39 exhibiting disease progression. Our analysis revealed a significant elevation in LRP1BSS among MGUS patients who eventually progressed to MM compared to those who did not (*P* = 1e−3, Figure 7A). As shown in Figure 7B, with LRP1BSS increasing, there is a corresponding escalation in the proportion of MGUS patients advancing to MM, particularly notable among the highest quintile (top 20%) of patients. Notably, the incidence of progression in this high LRP1BSS cohort was double that observed in the cohort with lower LRP1BSS levels (*P* = 0.03, Figure 7C).

## Discussion

*TP53* mutations are known to impact MM prognosis, with the *TP53* pathway being a target in MM and other cancers. Our study demonstrates that a TP53 signature based on transcriptomic genetic variability offers superior prognostic accuracy over models relying solely on *TP53* mutations or clinical factors. Crucially, we found that pathway regulation involving *LRP1B* is closely associated with MM progression and prognosis. This pathway can predict outcomes, TIME infiltration, drug responses, and the transition from MGUS to MM.

The role of *LRP1B*’s downstream regulatory pathways in MM has been less explored. *LRP1B* is considered a putative tumor suppressor^59-61^ and is involved in the regulation of the Wnt/β-catenin/TCF signaling pathway.^62^ In the context of MM, the study by Corre et al^63^ identified *LRP1B* as one of the most frequently mutated genes in patients with MM (12% of the patients). Furthermore, Vikova et al^64^ suggested that *LRP1B* is a novel mutated gene with potential significance in MM biology. Despite these findings, *LRP1B* is not widely recognized as a prognostic marker in MM. Our results support this by showing no significant correlation between *LRP1B* mutations and OS in patients with MM (Figure 1A). However, the overall impact of *LRP1B* pathways provides richer prognostic information, suggesting potential targets for therapeutic interventions. To further investigate, we calculated the mutation rate per base for each of the 762 patients using the CoMMpass dataset. By considering each gene’s length, we determined the expected number of mutations for each gene. The mutation enrichment ratio was then calculated by dividing the observed mutation count for each gene by its expected count, comparing each gene’s actual mutation count to the expected random mutation rate. The enrichment ratio for *LRP1B* is 2.2 (66 observed vs 30.2 expected). Although this is lower than *TP53* (8.6, 43/5.0) and *BRAF* (9.7, 52/5.4), it is higher than long genes with near-random mutation ratios like *TTN* (0.96, 192/199.7), *MUC16* (1.19, 96/80.1), and *SYNE1* (1.05, 53/50.7). This higher enrichment ratio for LRP1B indicates that *LRP1B* is indeed highly mutated in MM, rather than being subject to random mutations. We also analyzed the dN/dS ratio^65^ and found that the dN/dS ratio of *LRP1B* is 3.5 ((40 missense + 1 missense splice + 3 stop gain + 1 frameshift)/13 synonymous) in 762 patients, indicating potential selection pressure in MM. These results indicate that, although LRP1B has not yet been reported as a driver gene in MM, it has potential biological significance in MM. The LRP1BSS provides a potential prognostic and progression predictive tool that can characterize this significance.

*LRP1B*’s interaction with the MM TIME may explain part of its prognostic value. By analyzing LRP1BSS in CD138+ and WBM gene expressions, we observed how *LRP1B* pathway disturbances affect granulocyte and macrophage dynamics within TIME. Higher granulocyte infiltration, associated with better MM prognosis,^29^ and increased macrophage activity suggest complex interplays within TIME that could mediate MM progression. Specifically, the proliferation of oncogenic and anti-tumorigenic macrophage types in TIME suggests that the activities of M2 and M0 macrophages may diminish the anticancer effects of increased M1 macrophages.

Additionally, high LRP1BSS correlates with enhanced activities in pathways like the proteasome, spliceosome, and oxidative phosphorylation, as well as the citrate cycle/TCA cycle and protein export. Bortezomib, a proteasome inhibitor, leads to the accumulation of misfolded or unfolded proteins in the endoplasmic reticulum (ER), increasing ER stress and ultimately resulting in limited proliferation or apoptosis.^66,67^ Patients with MM who exhibit restored proteasome activity demonstrate resistance to bortezomib.^54^ Furthermore, previous studies suggest that inhibiting protein export reduces bortezomib resistance.^52,53^ Recent research proposes that interference with the spliceosome is an unidentified mechanism of PI action in MM, highly associated with PI resistance.^55^ Additionally, an increase in the TCA cycle and oxidative phosphorylation is widely regarded as a core feature of bortezomib-resistant MM cells.^56-58^ Therefore, patients with higher LRP1BSS may increase proteasome, protein export, and slicing activities, thereby alleviating ER stress and countering the efficacy of bortezomib treatment. They also exhibit higher energy metabolism pathways to respond to stress.

Overall, our research shows that genetic signature scores from TP53 and LRP1B pathways predict MM prognosis accurately. These scores offer new prognostic details beyond usual clinical factors, allowing for broader patient stratification. LRP1BSS reveals granulocyte and macrophage infiltration and resistance to bortezomib, aiding in therapeutic decision-making for all stages of MM. It also helps assess early MM stages, providing a tool for monitoring MGUS patients.

## Supplementary material

Supplementary material is available at *The Oncologist* online.

oyae244_suppl_Supplementary_Material

## Data Availability

All data generated in this study, including gene signatures, signature scores, and the analytical codes, are available at the following repository: https://github.com/JRLi/MM_gene_signatures. For the MMRF-CoMMpass study, the expression profiles and SNV data were available at https://portal.gdc.cancer.gov/projects/MMRF-COMMPASS, and the clinical data was available at the NCBI dbGaP under the phs000748.v7.p4. The expression and clinical data of other studies were available at the NCBI GEO under the GSE136400, GSE24080, GSE9782, GSE19784, GSE6477, GSE5900, and GSE235356.
