# Supplementary material for: Characterization of driver mutations identifies gene signatures predictive of prognosis and treatment sensitivity in multiple myeloma
Source: Oncologist. 2024 Sep 9;29(11):e1552–64. doi: 10.1093/oncolo/oyae244 (PMC11639189; doi:10.1093/oncolo/oyae244)
Supplement: oyae244_suppl_Supplementary_Material [file oyae244_suppl_supplementary_material.pdf]

Supplementary Figure 1

A

Signature	Dataset	No. of patient	HR (95% CI)	P-value
TP53SS	MAQC	558	1.48 (1.31 to 1.66)	6e-11
	UAMS	284	1.31 (1.10 to 1.57)	0.002
	HOVON65	280	1.36 (1.17 to 1.57)	6e-05
	APEX	242	1.51 (1.13 to 2.00)	0.005
LRP1BSS	MAQC	558	1.34 (1.17 to 1.53)	1e-05
	UAMS	284	1.59 (1.39 to 1.83)	5e-11
	HOVON65	280	1.32 (1.15 to 1.53)	1e-04
	APEX	242	1.50 (1.14 to 1.98)	0.004

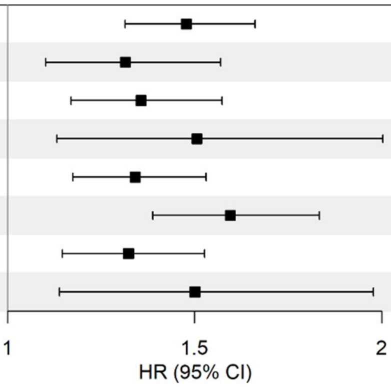

Figure S1. The prognostic prediction results of TP53SS and LRP1BSS in 4 independent datasets. (A) The prognosis evaluation of progression-free (PFS) or event-free survival (EFS) with the TP53 signature score (TP53SS) and LRP1B signature score (LRP1BSS) using 4 independent datasets, the TP53SS and LRP1BSS were predictive in all datasets.

Supplementary Figure 2

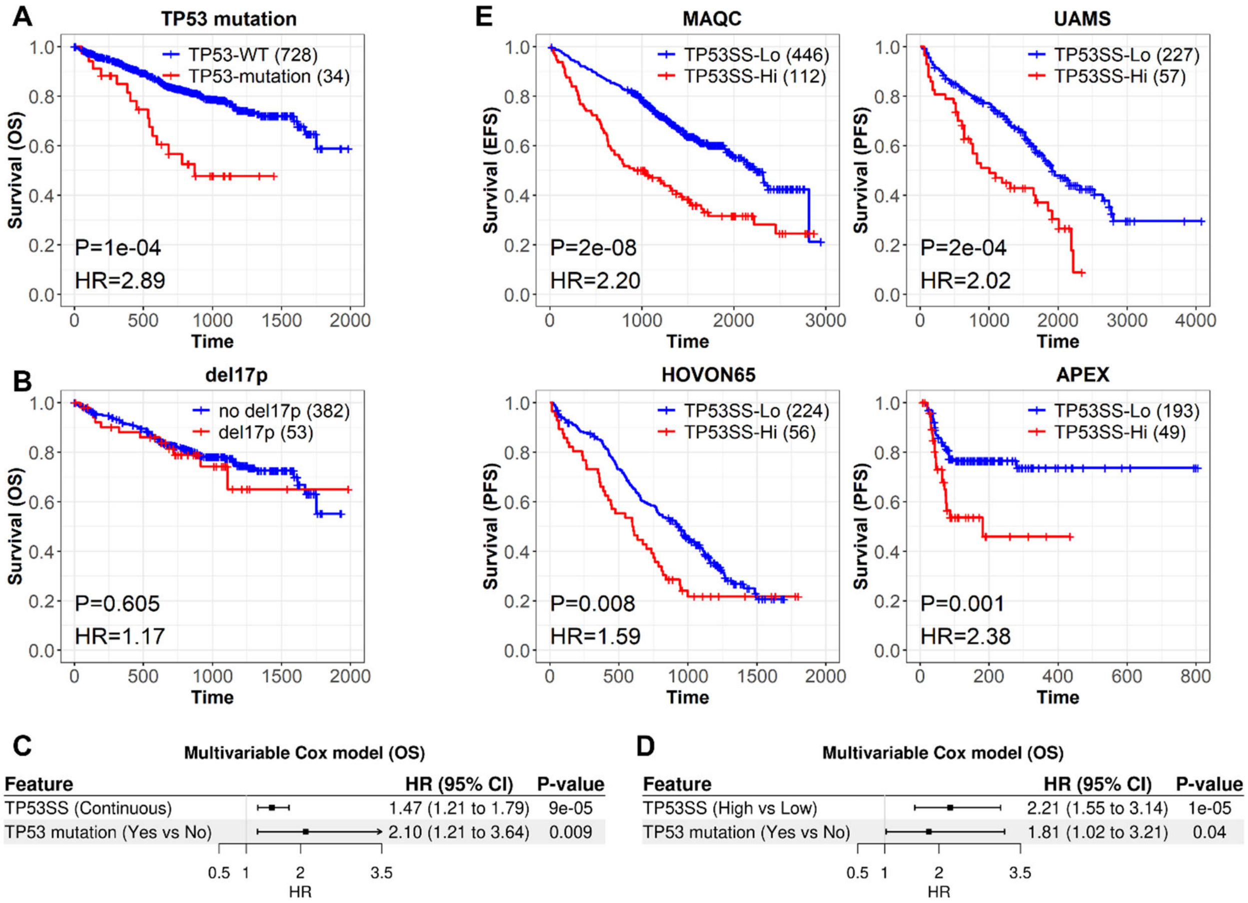

Figure S2. **The TP53SS is prognosis-predictive for MM patients. (A)** Overall survival curves of MM patients with *TP53* nonsynonymous mutation versus those with wild-type *TP53* in the CoMMpass dataset. MM patients with *TP53* nonsynonymous mutations have a significantly poorer prognosis. **(B)** Overall survival curves of for MM patients with cytogenetic abnormality del17p versus those without del17p in the CoMMpass dataset. MM patients with del17p have no different prognosis compared with those without del17p. **(C)** Prognostic prediction results of OS for MM in the CoMMpass dataset using continuous TP53SS and TP53 mutation status in the multivariable Cox regression. **(D)** Prognostic prediction results of OS for MM in the CoMMpass dataset using binary TP53SS (High vs Low) and TP53 mutation status in the multivariable Cox regression. **(E)** Event-free or progression-free survival curves of MM patients with higher TP53SS versus those with lower TP53SS in the MAQC, UAMS, HOVON65, and APEX datasets. MM patients with higher TP53SS have a significantly poorer prognosis.

Supplementary Figure 3

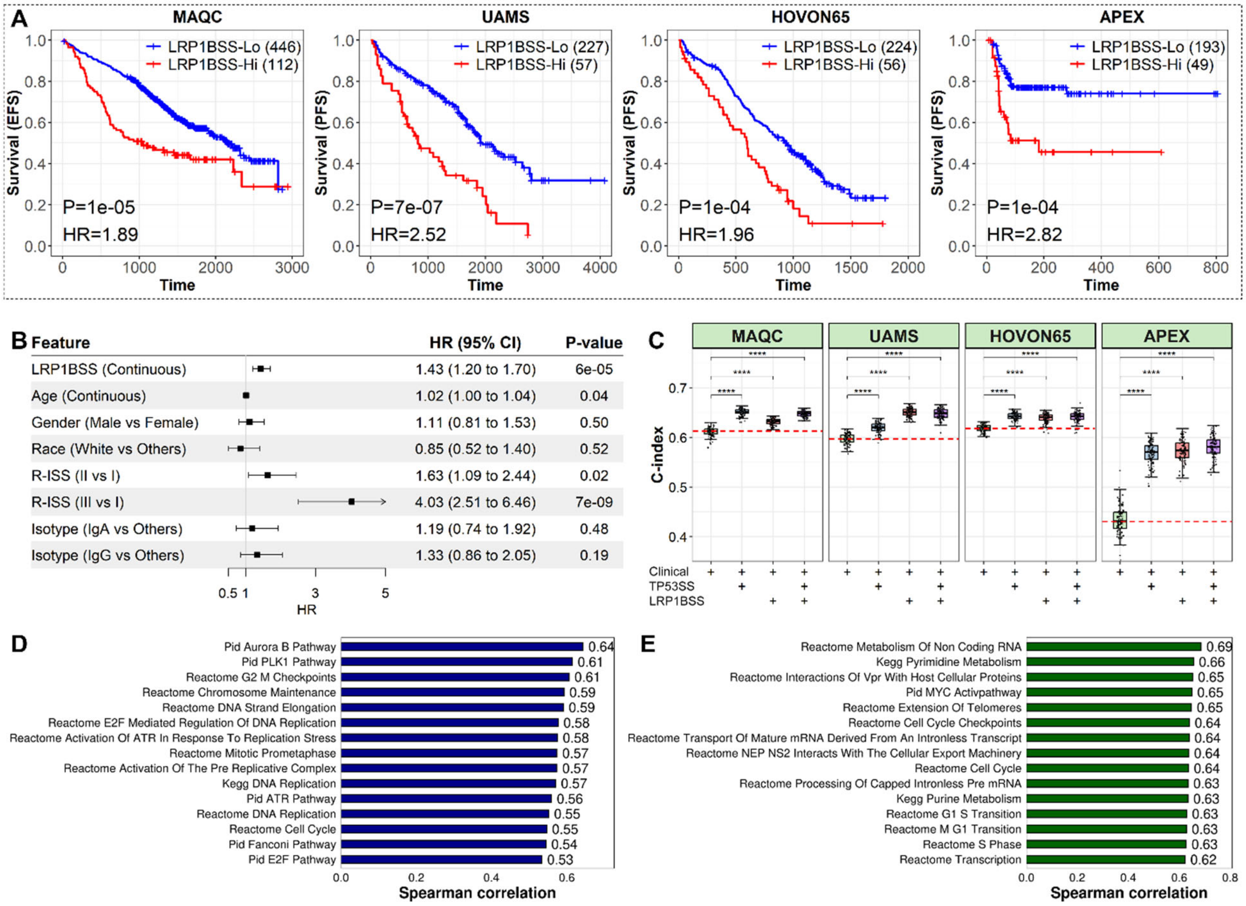

Figure S3. The LRP1BSS predicts the prognosis of MM patients in addition to the TP53SS. (A) Progression-free/Event-free survival curves of MM patients with higher LRP1BSS versus those with lower LRP1BSS in the MAQC, UAMS, HOVON65, and APEX datasets. MM patients with higher LRP1BSS have a significantly poorer prognosis. **(B)** The result of multivariable-adjusted Cox proportional hazards (PH) models of overall survival with age, gender, race, isotype, R-ISS stage system, and LRP1BSS in the MAQC dataset. **(C)** The C-index of the Cox PH prognosis model of EFS/PFS using the available clinical factors, clinical factors with TP53SS, clinical factors with LRP1BSS, and clinical factors with TP53SS and LRP1BSS in 4 independent datasets. ****: P < 0.0001. **(D)** Canonical pathways with significant positive correlation between GSVA scores and TP53SS. **(E)** Canonical pathways with significant positive correlation between GSVA scores and LRP1BSS.

Supplementary Figure 4

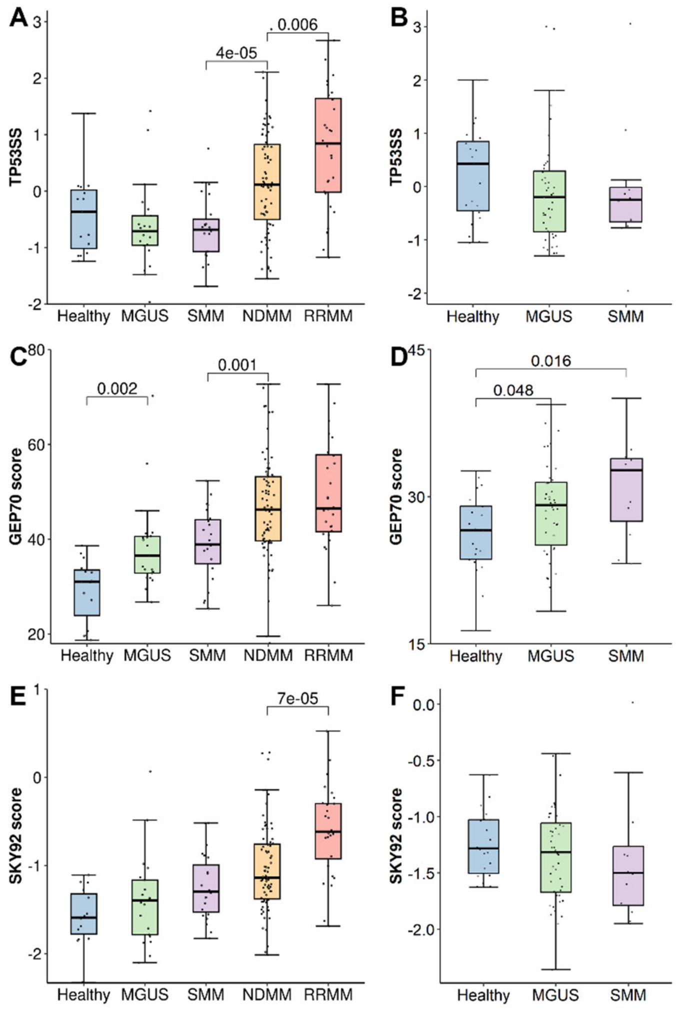

Figure S4. The TP53SS, GEP70 score, and SKY92 score between development stages of MM. (A) The TP53SS between healthy controls, patients with monoclonal gammopathy of unknown significance (MGUS), smoldering multiple myeloma (SMM), newly diagnosed multiple myeloma (NDMM), and relapsed or refractory multiple myeloma (RRMM) in the Mayo dataset. **(B)** The TP53SS between healthy controls, patients with MGUS, and patients with SMM in the Zhan dataset. **(C)** The GEP70 score between healthy controls, patients with MGUS, SMM, NDMM, and RRMM in the Mayo dataset. **(D)** The GEP70 score between healthy controls, patients with MGUS, and patients with SMM in the Zhan dataset. **(E)** The SKY92 score between healthy controls, patients with MGUS, SMM, NDMM, and RRMM in the Mayo dataset. **(F)** The SKY92 score between healthy controls, patients with MGUS, and patients with SMM in the Zhan dataset.

Supplementary Figure 5

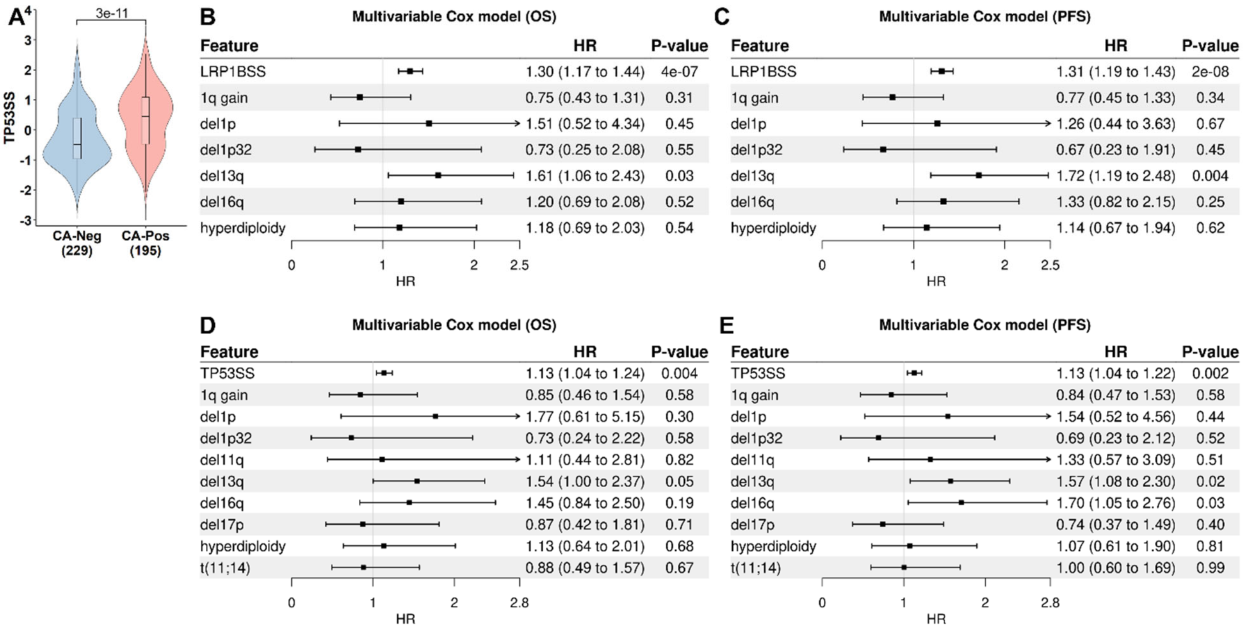

Figure S5. The TP53 and LRP1BSS provide additional prognosis information after adjusting their related CAs. (A) TP53SS between MM patients with and without cytogenetic abnormalities (CAs). Patients with CAs have higher TP53SS. **(B)** Multivariable Cox regression of OS using LRP1BSS and related CAs. LRP1BSS provided most significant prognostic information. **(C)** Multivariable Cox regression of PFS using LRP1BSS and related CAs. LRP1BSS provided most significant prognostic information. **(D)** Multivariable Cox regression of OS using TP53SS and related CAs. TP53SS provided most significant prognostic information. **(E)** Multivariable Cox regression of PFS using TP53SS and related CAs. TP53SS provided most significant prognostic information.

Supplementary Figure 6

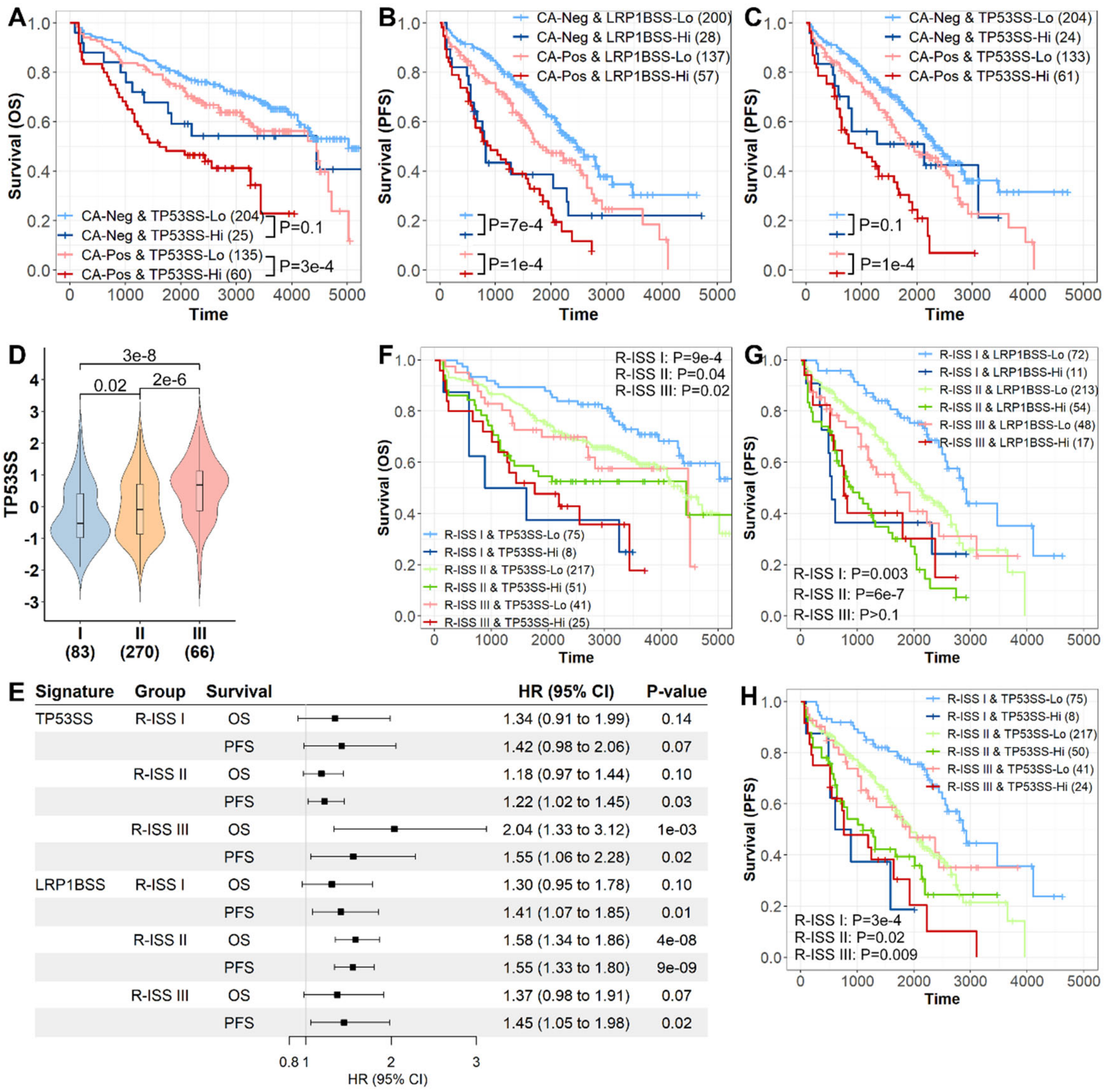

Figure S6. The TP53 and LRP1BSS provide additional prognosis information beyond the cytogenetic abnormalities and R-ISS. (A) Overall survival (OS) curves of MM patients with high or low TP53SS in the groups of patients with any CA (CA-Pos) or without any CA (CA-Neg). Patients with higher TP53SS have poorer prognoses only in the CA-Pos group. **(B)** Progression-free survival (PFS) curves of MM patients with high or low LRP1BSS in the CA-Pos or CA-Neg. Patients with higher LRP1BSS have poorer prognoses in both groups. **(C)** PFS curves of MM patients with high or low TP53SS in the CA-Pos or CA-Neg. Patients with higher TP53SS have poorer prognoses only in the CA-Pos group. **(D)** TP53SS between MM patients with the R-ISS stage I, II, and III. Patients with higher R-ISS have higher TP53SS. **(E)** Continuous values of TP53SS and LRP1BSS predict the prognosis of OS or PFS in patients with the R-ISS I, II, or III. **(F)** OS curves of MM patients with higher and lower TP53SS that belong to R-ISS I, II, and III. Patients with higher TP53SS had poor outcomes in R-ISS I, II, and III. **(G)** PFS curves of MM patients with higher and lower LRP1BSS that belong to R-ISS I, II, and III. Patients with higher LRP1BSS had poor outcomes in R-ISS I and II but not in R-ISS III. **(H)** PFS curves of MM patients with higher and lower TP53SS that belong to R-ISS I, II, and III. Patients with higher TP53SS had poor outcomes in R-ISS I, II, and III. All results are based on the UAMS dataset. Significant level was calculated using the Wilcoxon rank sum test.

Supplementary Figure 7

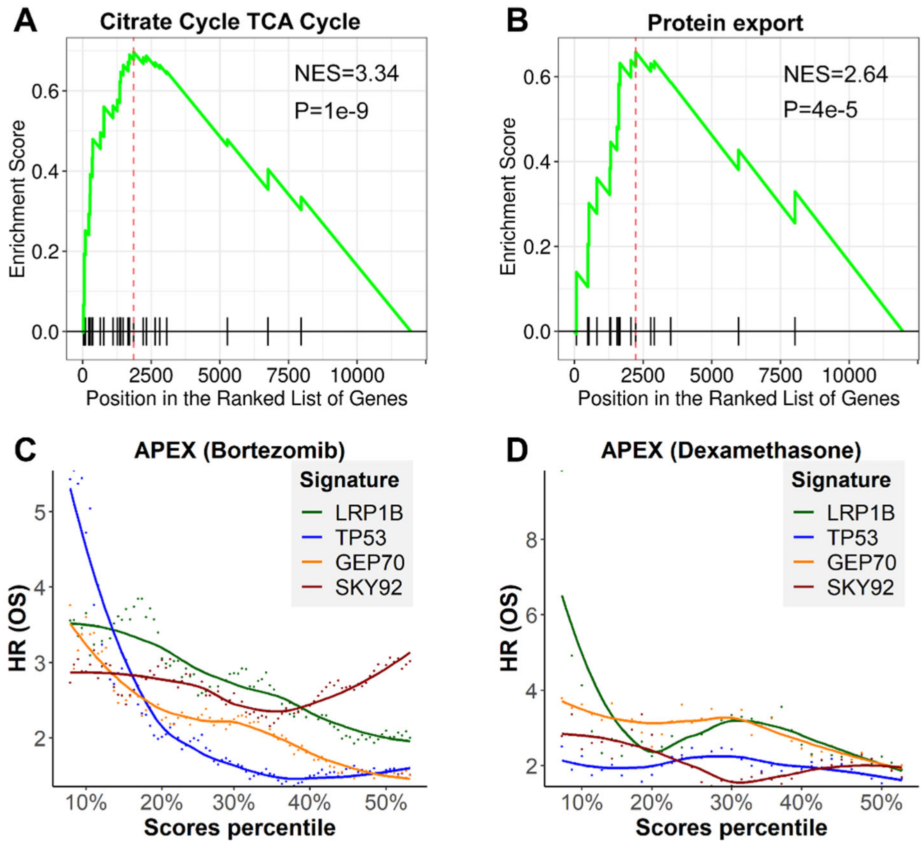

Figure S7. The LRP1BSS are related to pathways associated with the proteasome inhibitor. (A) The rank 14th significantly enriched KEGG pathways, Citrate Cycle TCA Cycle, using the GSEA analysis. **(B)** The rank 28th significantly enriched KEGG pathways, Protein Export, using the GSEA analysis. **(C)** RMM patients treated with bortezomib selected by LRP1BSS (top f% with highest scores) showed a higher hazard ratio (HR) relative to the rest patients than those selected by the other three signatures. **(D)** RMM patients treated with dexamethasone selected by LRP1BSS (top f% with highest scores) showed a higher hazard ratio (HR) relative to the rest patients than those selected by the other three signatures

Supplementary Table S1. Summary of datasets used in this study

Study	Name	Patients with survival information	Patient type	Biospecimen	Gene expression data	Data Source	Applications and notes in this study
**CoMMpass**	MMRF-CoMMpass	762	NDMM	BM CD138+ cell	RNA-seq	NCI GDC	Training dataset
**MAQC**	Danziger et al.	558	NDMM	BM CD138+ cell, WBM	Affy HGU133 Plus 2.0	GSE136400	Validation dataset
**UAMS**	MAQC-II	424	NDMM	BM CD138+ cell	Affy HGU133 Plus 2.0	GSE24080	Validation dataset. In prognostic analyses, use 286 patients who did not overlap with MAQC. In TIME, use 401 patients with paired WBM data
**HOVON65**	HOVON65	280	NDMM	BM CD138+ cell	Affy HGU133 Plus 2.0	GSE19784	Validation dataset
**APEX**	APEX phase 3 trial	264	RMM, 188 treated with Bortezomib (169 with response information) and 76 treated with Dexamethasone (70 with response information).	BM CD138+ cell	Affy HGU133 A	GSE9782	Validation dataset
**Mayo**	Mayo Clinic		15 healthy controls, 21 MGUS, 23 SMM, 75 NDMM, and 28 RRMM	BM CD138+ cell	Affy HGU133 A	GSE6477	MM development
**Zhan**	Zhan et al.		22 healthy controls,44 MGUS,12 SMM	BM CD138+ cell	Affy HGU133 Plus 2.0	GSE5900	MM development
**Sun**	Sun et al.		319 stable MGUS, 39 progression MGUS	BM CD138+ cell	Affy HGU133 Plus 2.0	GSE235356	MGUS progression

Supplementary table S2. Frequently mutated genes in MM and their association with cancer

Gene	# nonsynonymous/indel in CoMMpass	COSMIC CGC
IGHV2-70	453	N
IGLV3-1	271	N
KRAS	173	**Y**
NRAS	147	**Y**
TTN	89	N
IGKV4-1	82	N
FAM46C	78	N
DIS3	73	N
IGHM	71	N
IGHV1-69-2	71	N
IGLL5	67	N
MUC16	66	**Y**
BRAF	51	**Y**
IGLV4-60	50	N
TRAF3	50	N
FAT4	46	**Y**
RYR2	45	N
IGLV3-25	44	N
PCLO	42	N
IGHV1-69	42	N
LRP1B	42	**Y**
USH2A	39	N
DUSP2	36	N
RYR1	35	**Y**
DNAH5	35	N
FAT3	35	N
CSMD3	35	**Y**
ZFHX4	35	N
FAT1	34	**Y**
TP53	34	**Y**
FSIP2	32	N
HIST1H1E	31	N
IGLV5-45	31	N
